# Chemerin as a novel non-invasive serum marker of intrahepatic lipid content in obese children

**DOI:** 10.1186/s13052-014-0084-4

**Published:** 2014-11-17

**Authors:** Monika Kłusek-Oksiuta, Irena Bialokoz-Kalinowska, Eugeniusz Tarasów, Malgorzata Wojtkowska, Irena Werpachowska, Dariusz Marek Lebensztejn

**Affiliations:** Department of Pediatrics, Gastroenterology and Allergology, Medical University of Bialystok, 17 Waszyngtona St., Bialystok, 15-274 Poland; Department of Pediatrics and Developmental Disorders, Medical University of Bialystok, Bialystok, Poland; Department of Radiology, Medical University of Bialystok, Bialystok, Poland; Department of Radiology, University Teaching Children’s Hospital, Bialystok, Poland

**Keywords:** Adipokines, Chemerin, Omentin, Vaspin, Intrahepatic lipid content, NAFLD, Children

## Abstract

**Background:**

Ectopic hepatic lipid accumulation is closely related to the development of insulin resistance, which is regarded as one of the most significant risk factors of non-alcoholic fatty liver disease (NAFLD). The current study has shown that fat tissue constitutes an important endocrine organ with its own production and metabolism of many biologically active substances, among which adipokines play an important role. Classic adipokines (e.g. leptin, adiponectin, resistin) are fat-derived hormones which serum level is altered in patients with NAFLD. The role of novel adipokines in the pathomechanism of this disease is not clear. Therefore, the aim of our study was to evaluate the serum concentrations of chemerin, omentin and vaspin in obese children with NAFLD.

**Methods:**

Forty-five obese children, aged 7–17 years old, were admitted to our Department with suspected liver disease (hepatomegaly, and/or ultrasonographic liver brightness, and/or increased ALT activity). Viral hepatitides, as well as autoimmune and metabolic liver diseases were excluded. Fasting serum levels of chemerin, omentin and vaspin were determined. The grade of liver steatosis in ultrasound was graded according to Saverymuttu. ^1^HMR spectroscopy was performed with a 1.5 T scanner and with PRESS sequencing.

**Results:**

Fatty liver was confirmed in 39 children by ultrasound and in 33 patients by ^1^HMRS (19 of them also had increased ALT activity /NAFLD/). Chemerin and vaspin levels were significantly higher in children with NAFLD compared to the control group (n = 30). The concentration of chemerin was significantly higher in children with advanced liver steatosis compared to non-hepatopathic patients (p = 0,02). Significant positive correlations were found between the total liver lipids in ^1^HMRS and chemerin (r = 0,33; p = 0,02) and vaspin (r = 0,4; p = 0,006). The ability of serum chemerin (cut-off = 190 ng/ml, Se = 75%, Sp = 58%) to differentiate children with fatty liver in ^1^HMRS from those without steatosis was significant (AUC = 0,7, p = 0,04). Omentin and vaspin did not allow a useful prediction to be made.

**Conclusion:**

Chemerin seems to be the most suitable non-invasive biomarker in predicting both intrahepatic lipid content in obese children and advanced liver steatosis in children with NAFLD.

## Background

Due to the increase in obesity prevalence observed throughout the world during the last few decades, nonalcoholic fatty liver disease (NAFLD) has become the most common cause of chronic liver disease, both in adults and children [1]. NAFLD is commonly associated with visceral obesity, dyslipidemia and insulin resistance, which constitutes the specific manifestation of metabolic syndrome [2]. Results from many studies have confirmed that both metabolic syndrome and NAFLD contribute to an acceleration of atherosclerosis development. Moreover, individuals with NAFLD are at much higher risk of developing cardiovascular disease (CVD) compared to individuals in the general population [3,4]. Additionally, fat distribution seems to be a better predictor of (CVD) compared to obesity alone [5]. It has been proven that not only visceral obesity, but also ectopic fat accumulation in the liver is strictly associated with the development of insulin resistance and increase of cardiometabolic risk [6,7]. The current study has shown that fat tissue constitutes an important endocrine organ with its own production and metabolism of many biologically active substances, among which adipokines play an important role [8]. These proteins, produced and secreted by fat tissue, take part in many physiological processes and are responsible for such pathological changes associated with obesity as insulin resistance, atherosclerosis, hypertension and NAFLD. Lately, it has been suggested that so called “novel” adipokines (chemerin, omentin, vaspin) might have a strong relationship with obesity, insulin resistance, atherosclerosis, metabolic syndrome and fatty liver. It has been shown in a few studies, comprised of adult populations, that some of these adipokines are displayed in higher concentrations in NAFLD patients compared to the control. They may have an important role in progression from simple steatosis to inflammation and fibrosis. Moreover, a correlation was found between their serum concentrations and the morphological features of NAFLD (hepatocytes ballooning, NAS index, fibrosis) [9-12]. However, an accurate assessment of the role of the above mentioned adipokines is still lacking in the pediatric population. It should be emphasized that the studies performed in children may be more convincing compared to those in adults, due to the existence of far fewer confounding factors in such a population and the fact that disease is usually less advanced. Therefore, the objective of this study was to evaluate serum chemerin, omentin and vaspin concentrations in children with NAFLD as potential markers of ectopic lipid accumulation in the liver.

## Methods

This prospective study included a group of 45 consecutive obese children (35 boys and 10 girls) aged 7–17 years old (mean 13 years old) admitted to our Department in 2011–2012 due to suspected liver pathology (hepatomegaly and/or elevated alanine aminotransferase/ALT/ and/or fatty liver features in ultrasound/US/examination), as previously described [13]. Informed consent was obtained from all patients’ parents. The protocol was approved by the bioethics committee of the Medical University of Bialystok.

Viral infection due to hepatitis C virus (HCV) and due to hepatitis B virus (HBV), autoimmune hepatitis, chosen metabolic liver diseases (Wilson disease, alpha-1-antitrypsin deficiency, cystic fibrosis) and drug-induced liver injury (DILI) were excluded in all children. Moreover, children with diabetes and those receiving drugs that could affect lipid or carbohydrate metabolism were excluded from this study.

All subjects underwent physical examination with anthropometric measurements (body mass index/BMI/, which was calculated from measurements of height and weight, and waist circumference). Routine blood chemistry analyses (ALT, gamma glutamyltransferase/GGT/, total cholesterol, high-density lipoprotein/HDL/ and low-density lipoprotein/LDL/cholesterol, triglycerides/TG/, glucose and insulin) were performed using standard methods. Homeostatic model assessment –insulin resistance/HOMA-IR/ was calculated according to the formula described by Matthews et al. [14].

Fasting serum adipokine concentration (chemerin, omentin, vaspin) was assessed using the commercial ELISA kits (BioVender, Brno, Czech Republic). The control group was comprised of 30 non-obese children without any somatic organ pathology. Blood samples were immediately centrifuged and stored in −80°C until further processing. The intra- and inter-assay CVs of chemerin ranged from 5.1 to 7.0% and 6.9 to 8.3% respectively. The intra- and inter-assay CVs of vaspin ranged from 6.5 to 8.7% and 5.8 to 9.5% respectively. The intra- and inter-assay CVs of omentin α ranged from 3.2 to 4.1% and 4.4 to 4.8% respectively. All assays were conducted according to the manufacturer’s instructions.

Liver ultrasound examination was performed with General Electric Voluson E8, convex sonde 3–5 MHz; the intensity of steatosis was assessed using a four-grade scale (0–3) as outlined by Saverymuttu et al. [15]. Advanced liver steatosis was defined as a score >1. Steatosis grade was assessed in a blinded fashion by the same radiologist without knowledge of the patients’ laboratory or clinical data. ^1^HMR spectroscopy was performed with a 1.5 T scanner (Picker Eclipse) and with PRESS sequencing. Total intrahepatic lipid content was assessed in relative units (r.u.) in comparison to unsuppressed water signal. Voxele’s size 3×3×3 cm (27 cm^3^) was localized in the right liver lobe in the manner that it would not comprise the vessels and bile ducts [16].

Thirty nine children (87%) had liver steatosis in ultrasound examination (group I); 19 of them also had an increased serum ALT activity (group Ia – NAFLD). Six children (13%) had neither liver brightness in ultrasound nor increased ALT activity (group II – non-hepatopathic obese children) (Table 1).

**Table 1:** **The characteristics of groups of examined obese children**

**Parameter**	**Group I (n = 39) median; Q**_**1**_ **- Q**_**3**_	**Group Ia (n = 19) median; Q**_**1**_ **- Q**_**3**_	**Group II (n = 6) median; Q**_**1**_ **- Q**_**3**_	**p I vs II**	**p Ia vs II**
Chemerin (ng/ml)	228.5; 178.4-251	229.4; 192.3-251.0	186; 159.5-201.8	0.06 (NS)	0.028
Vaspin (ng/ml)	0.09; 0.03-0,21	0.11; 0.036-0.42	0.07; 0.02-0.16	NS	NS
Omentin (ng/ml)	194.5; 156.4-300.8	233.6; 163.0-335.1	201.5; 189.6-229.3	NS	NS
Age (years)	14; 12-16	14; 13-16	11; 10-14	NS	NS
BMI (kg/m^2^)	27.4; 26.0-32.2	28.8; 26.8-32.5	25.4; 25.4-28.2	NS	0,02
Waist (cm)	97.5; 92-105	101; 96-107	85.5; 81-98	0.05	0.01
ALT (IU/l)	37; 27-57	57; 50-84	17.5; 15-28	0.008	0.0002
GGT (IU/l)	24; 18-31	30; 23-45	13.5; 10-18	0.006	0.001
Cholesterol (mg/dl)	178; 143-210	180; 139-220	141.5; 130-182	NS	NS
HDL-cholesterol (mg/dl)	44; 39-54	42; 39-51	43; 40-50	NS	NS
LDL-cholesterol (mg/dl)	110; 82-130	108; 81-143	77; 73-105	NS	NS
TG (mg/dl)	111; 68-152	140; 64-182	87.5; 75-126	NS	NS
Glucose (mg/dl)	92; 89-97	94; 85-101	93; 89-96	NS	NS
Insulin (μIU/ml)	13.3; 10.3-17.4	15.5; 10.3-21.0	14; 9.6-18.4	NS	NS
HOMA-IR	3.08; 2.39-3.88	3.67; 2.42-4.49	3.4; 2.1-4.0	NS	NS
Lipids ^1^HMRS (r.u.)	107; 79-163	139; 102-179	19.5; 14-38	0.006	0.004

### Statistical analysis

The serum concentrations of adipokines, other biochemical tests and anthropometric parameters were expressed as median; 25–75 quartile. The statistical analysis was performed with the Mann–Whitney two-sample test for nonparametric data. The relationship between biochemical tests was analyzed by the Spearman rank-correlation test for non-parametric data. The tests were considered statistically significant at p < 0.05. Logistic regression analysis was performed using IBM SPSS Statistics 20.0.

The receiver operating characteristics (ROC) analysis was used to calculate the power of the assays to detect liver steatosis. The comparison of the area under curve (AUC) was performed using a two-tailed p-test, which compares the AUC to the diagonal line of no information (AUC 0.5) [17].

## Results

There were significantly higher BMI and waist circumferences, activity of ALT and GGT and intrahepatic lipid content in ^1^HMRS in children with NAFLD compared to non-hepatopathic patients (Table 1).

### Serum concentration of chemerin, vaspin and omentin

The serum levels of chemerin (229.4; 192.3-251.0 ng/ml) and vaspin (0.11; 0.036-0.42 ng/ml) in NAFLD children were significantly higher than in lean controls (113.5; 98.2-130.5, 0.04; 0.02-0.11 ng/ml respectively) (p < 0.0001; p = 0.019 respectively). There were no significant differences in omentin in both groups; however, a lower value of omentin was found in NAFLD patients (233.6; 163.0-395.1 vs 311.9; 583.3-738.6 ng/ml). There was a significant positive correlation of chemerin with: GGT (r = 0.3; p = 0.03), an intensity of the hepatic steatosis in US (r = 0,3; p = 0,04) and intrahepatic lipid content in HMRS (r = 0.33; p = 0.02). Vaspin correlated with ALT and GGT (r = 0.33; p = 0.02, r = 0.34; p = 0.02, respectively), intensity of the hepatic steatosis in US (r = 0.3; p = 0.04) and intrahepatic lipid content in ^1^HMRS (r = 0.4; p = 0.0006). Omentin correlated only with total cholesterol (r = 0.3; p = 0.04) and LDL-cholesterol (r = 0.3; p = 0.05).

Nineteen children had advanced liver steatosis (grade 2 or 3) and 6 had no steatosis according to the Saverymuttu scale [15]. The concentration of chemerin was significantly higher in children with advanced liver steatosis compared to non-hepatopathic patients (p = 0.02) (Table 2).

**Table 2:** **The characteristics of subgroups of children with advanced liver steatosis in US (n = 19) and without liver steatosis in US (n = 6)**

**Parameter**	**USG - 0 (n = 6) median; Q**_**1**_ **- Q**_**3**_	**USG - 2 or 3 (n = 19) median; Q**_**1**_ **- Q**_**3**_	**p**
Chemerin (ng/ml)	186; 159.5-201.8	229.4; 196.6-254.6	0.02
Vaspin (ng/ml)	0.07; 0.02-0.16	0.18; 0.04-0.4	NS
Omentin (ng/ml)	201.5; 189.6-229.3	193.9; 166-279	NS
Age (years)	11; 10-14	14; 12-16	NS
BMI (kg/m^2^)	25.4; 25.4-28.2	27.9; 27–32.5	0.04
Waist (cm)	85.5; 81-98	102; 94-107	0.01
ALT (IU/l)	17.5; 15-28	46; 33-72	0.003
GGT (IU/l)	13.5; 10-18	27; 22-31	0.002
Cholesterol (mg/dl)	141.5; 130-182	177; 140-192	NS
HDL-cholesterol (mg/dl)	43; 40-50	40; 37-50	NS
LDL-cholesterol (mg/dl)	77; 73-105	95; 79-121	NS
TG (mg/dl)	87.5; 75-126	123; 82-182	NS
Glucose (mg/dl)	93; 89-96	95; 89-101	NS
Insulin (μIU/ml)	14; 9.6-18.4	13.1; 12.1-17.5	NS
HOMA-IR	3.4; 2.1-4.0	3.3; 2.7-4.1	NS
Lipids ^1^HMRS (r.u.)	19.5; 14-38	154; 102-181	0.001

### Diagnostic value of novel adipokines for identification of patients with liver steatosis confirmed in ^1^HMRS

We confirmed liver steatosis in 33 children in ^1^HMRS. Children with diagnosed liver steatosis in ^1^HMRS had higher levels of chemerin, ALT, triglycerides and an intensity of the hepatic steatosis in ultrasound examination (Table 3).

**Table 3:** **The comparative characteristics of obese children with diagnosed liver steatosis in 1HMRS(n = 33) and without liver steatosis in** ^**1**^ **HMRS (n = 12)**

**Parameter**	**Lack of liver steatosis in** ^**1**^ **HMRS (n = 12) median; Q**_**1**_ **-Q**_**3**_	**Liver steatosis in** ^**1**^ **HMRS (n = 33) median; Q**_**1**_ **–Q**_**3**_	**p**
Chemerin (ng/ml)	186; 159.8-227.8	228.7; 188.8-251.0	0.05
Vaspin (ng/ml)	0.09; 0.02-0.19	0.08; 0.04-0.21	NS
Omentin (ng/ml)	176.3; 136.9-239.1	201.5; 166.3-289.8	NS
Age (years)	14; 11–15.5	13; 12-15	NS
BMI (kg/m^2^)	28.1; 25.7-30.9	27.1; 25.6-32.0	NS
Waist (cm)	95.0; 89–103.5	97.0; 89.0-105.0	NS
ALT (IU/l)	36; 15–42.5	36.5; 27.0-56.5	0.05
GGT (IU/l)	19.5; 11.5-23.5	25.5; 18.0-30.5	NS
Cholesterol (mg/dl)	177; 137-198	178.5; 141.5-203.0	NS
HDL – cholesterol (mg/dl)	48.5; 43–51.5	42.5; 39-54	NS
LDL – cholesterol (mg/dl)	108; 74-136	108; 81-129	NS
TG (mg/dl)	84.5; 61–111.5	120; 86.5-165.5	0.02
Glucose (mg/dl)	92.5; 88.5-96.5	93; 89-97	NS
Insulin (μIU/ml)	13.5; 9.5-17.3	14.1; 11.8-17.5	NS
HOMA-IR	3; 2.03-3.96	3.3; 2.7-4.1	NS
Steatosis grade in US	1.0; 0.0-1.0	2.0; 1.0-2.0	0.0004

In the logistic regression analysis it was demonstrated that only chemerin, one out of three tested adipokines, may be useful in the differentiation of obese patients with confirmed hepatic steatosis in ^1^HMRS from patients without steatosis (OR-1.018, 95% CI 1.001-1.035, p = 0.044). Among other anthropometric and biochemical parameters evaluated in our study, only triglicerdes (OR-1.019, 95% CI 1.004-1.034, p = 0.012) presented the differentiation capacity for both conditions (Table 4).

**Table 4:** **Logistic regression analysis to differentiate of obese patients with confirmed hepatic steatosis in** ^**1**^ **HMRS from patients without steatosis**

**Parameter**	**B**	**OR**	**95% CI**	**p**
Chemerin	0.02	1.018	1.001-1.035	**0.044**
Vaspin	0.80	2.223	0.158-31.229	0.554
Omentin	0.00	1.004	0.994-1.015	0.404
ALT	0.01	1.014	0.973-1.056	0.514
GGT	0.02	1.020	0.960-1.083	0.522
Cholesterol	0.00	1.002	0.986-1.019	0.789
HDL-cholesterol	−0.02	0.976	0.920-1.035	0.417
LDL-cholesterol	0.00	0.998	0.979-1.018	0.863
TG	0.02	1.019	1.004-1.034	**0.012**
Glucose	0.02	1.019	0.935-1.112	0.664
Insulin	0.02	1.022	0.912-1.145	0.709
HOMA-IR	0.09	1.097	0.686-1.752	0.699
BMI	−0.04	0.965	0.814-1.143	0.680
Waist	0.01	1.012	0.958-1.068	0.676

**Figure 1:**
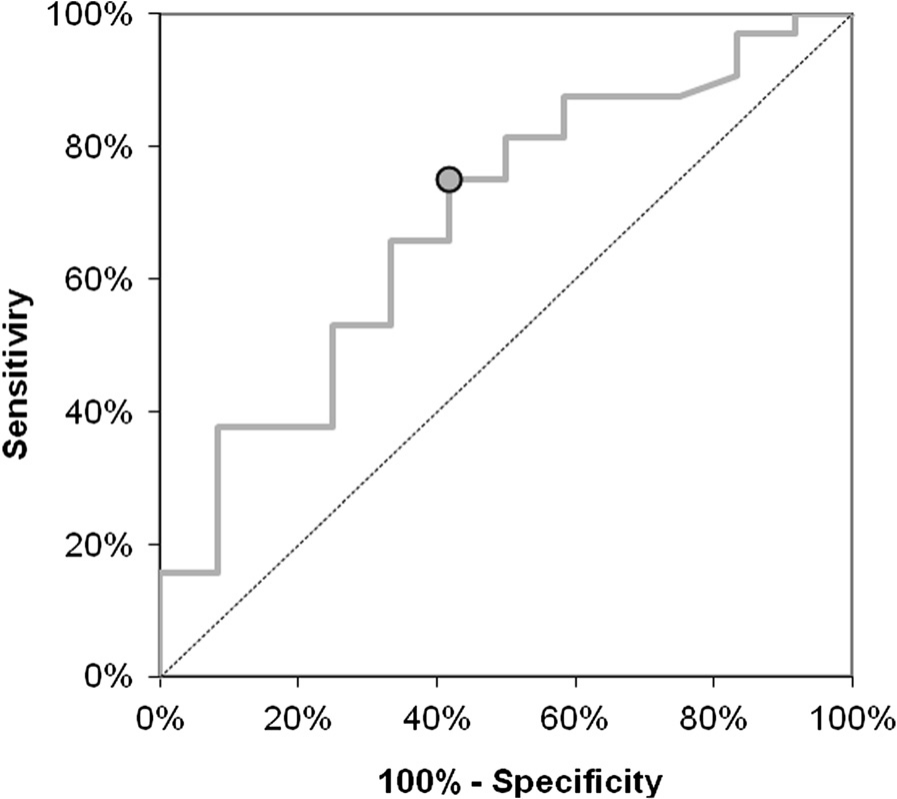
**ROC curve of ability of chemerin to detect children with liver steatosis in** ^**1**^ **HMRS.**

This finding was confirmed in ROC analysis. The ability of chemerin (cut-off = 190 ng/ml, Se = 75%, Sp = 58%) to differentiate the children with liver steatosis in ^1^HMRS from those without steatosis was significant (AUC = 0.7, p = 0.04). Omentin and vaspin did not allow a useful prediction to be made (Figure 1).

## Discussion

In our study, significantly higher serum chemerin and vaspin concentrations were found in obese children with NAFLD compared to the control. However, no significant differences in omentin serum concentration were observed in either group. This type of study is currently lacking in the pediatric population. Partially consistent results have been achieved in a few studies in adults with morphologically confirmed NAFLD, although the three novel adipokines (simultaneously examined in this study) were not assessed at the same time in any of them. Kukla et al. [9] and Sell et al. [18] have demonstrated higher serum chemerin concentration and Yilmaz et al. [19] have also found higher chemerin and omentin concentrations in patients with NAFLD compared to the control group. Genc et al. [20] have confirmed higher serum concentration of vaspin in patients with NASH compared to the control. No differences in chemerin concentration in patients with NAFLD and controls were found by Ye et Al. [21]; however, it should be noted that NAFLD was diagnosed on the basis of incomplete criteria, namely by stating the characteristics of the fatty liver in US examination. The different concentrations of evaluated adipokines in children with NAFLD compared to the healthy population reflect their implication in the pathogenesis of this disease.

However, the main objective of this study was to determine whether the examined novel adipokines can be considered as non-invasive biomarkers of ectopic, intrahepatic lipid accumulation in the liver. For the first time, we have shown a positive correlation between chemerin and vaspin concentration and the severity of steatosis and lipid content in the liver in imaging studies; however omentin did not correlate with the above parameters.

In a study with adults, only Chen et al. [22] confirmed the correlation between chemerin concentration and the severity of steatosis assessed by magnetic resonance, but those studies were performed on hemodialysis patients, not patients with NAFLD.

Genc et al. [20] showed no vaspin correlation with steatosis confirmed in a morphological study of the liver in patients with NAFLD. However, chemerin was not evaluated in this group. In our study, ectopic fat accumulation in the liver was assessed by using proton magnetic resonance spectroscopy (^1^HMRS), which is considered to be a reliable, non-invasive diagnostic method [23,24]. A liver biopsy, which is considered the “gold standard”, was not performed in our study. This procedure is invasive, may be associated with risk of complications, and the obtained morphological results would not change or influence the therapeutic procedure [25]. Therefore, the currently accepted criteria for liver biopsy in children, established by Roberts [26], indicate that a liver biopsy in children with suspected NAFLD should be performed only in very specific cases (suspected advanced disease process, the exclusion of co-existing diseases, in patients up to 10 years of age and with high serum ALT activity, and before initiating pharmacological intervention). The examined children in our study did not meet the criteria necessary to perform a liver biopsy.

To date, there is no data stating the predictive value of examined adipokines for the diagnosis of fatty liver in obese children. In the above analysis, we observed higher concentrations of chemerin in children with hepatic steatosis confirmed in ^1^HMRS compared to the group of obese children without liver pathology. This observation was confirmed in the ROC analysis: the ability of serum chemerin to differentiate children with fatty liver in ^1^HMRS from those without steatosis was significant. Omentin and vaspin did not allow a useful prediction to be made.

Furthermore, in our study we have shown that among the analyzed adipokines only chemerin differentiated children with advanced hepatic steatosis from the non-hepatopathic obese. Therefore, it seems that chemerin can be considered a suitable biomarker of intrahepatic lipid content in obese children.

Although the study performed using animal models to assess chemerin expression in hepatic tissue is not conclusive [27], a recent study in humans has confirmed a high expression of chemerin mRNA in hepatocytes [28]. Moreover, a correlation between necroinflammatory activity and chemerin concentration in the serum of adults with NAFLD has been shown [29]. Studies conducted in pediatric populations confirmed that chemerin may be regarded as a marker of systemic and vascular inflammation [30,31]. The above analysis has confirmed the correlation between chemerin and ALT activity, which may be regarded as an indicator of inflammatory processes in the liver. Thus, it seems that children with NAFLD who demonstrate high chemerin serum concentrations may not only present simple fatty liver, but also its progression to NASH.

Our work has several potential limitations. Besides the lack of morphological examination in the studied group, which is the best diagnostic tool for confirming NAFLD, as discussed above, a selection bias should be mentioned, since examined patients were recruited from a tertiary center, which focuses on pediatric hepatology. As a result of this, children with previously suspected pathology of the liver were referred to the center and included to the study group later. The relatively small sample size limits the generalizability of our findings.

However in conclusion, the obtained data allow us to suggest that chemerin plays a role in the pathogenesis of NAFLD in children. Chemerin seems to be the most suitable non-invasive biomarker in predicting both intrahepatic lipid content in obese children and advanced liver steatosis in children with NAFLD.
